# Endometrial Stromal Sarcoma: An Update

**DOI:** 10.3390/cancers17111893

**Published:** 2025-06-05

**Authors:** Giulio Ricotta, Silvio Andrea Russo, Anna Fagotti, Alejandra Martinez, Elodie Gauroy, Mathilde Del, Valentin Thibaud, Bataillon Guillaume, Gwenaël Ferron

**Affiliations:** 1 Surgical Oncology, Oncopole Claudius Regaud-Institut Universitaire du Cancer Toulouse Oncopole, 31100 Toulouse, France; silvioandrea.russo01@icatt.it (S.A.R.); alejandra.martinez@iuct-oncopole.fr (A.M.); elodie.gauroy@iuct-oncopole.fr (E.G.); mathilde.del@iuct-oncopole.fr (M.D.); ferron.gwenael@iuct-oncopole.fr (G.F.); 2 Gynecologic Oncology Unit, Department of Women, Child and Public Health Sciences, Fondazione Policlinico Universitario Agostino Gemelli IRCCS, 00168 Rome, Italy; anna.fagotti@policlinicogemelli.it; 3 Oncology, Oncopole Claudius Regaud-Institut Universitaire du Cancer Toulouse Oncopole, 31100 Toulouse, France; valentin.thibaud@iuct-oncopole.fr; 4 Anatomopathology, Oncopole Claudius Regaud-Institut Universitaire du Cancer Toulouse Oncopole, 31100 Toulouse, France; bataillon.guillaume@iuct-oncopole.fr; 5 INSERM CRCT19, Oncogenesis of Sarcomas, 31037 Toulouse, France

**Keywords:** endometrial stromal sarcoma, gynecologic oncology, uterine sarcoma

## Abstract

Endometrial stromal sarcoma (ESS) is a rare malignant tumor of uterine mesenchyme, accounting for 15–20% of uterine sarcomas. It is classified into low-grade (LG-ESS) and high-grade (HG-ESS) subtypes, each defined by distinct histopathological and molecular features. LG-ESS exhibits slow progression, resembling proliferative-phase endometrial stroma, with genetic alterations like JAZF1-SUZ12 fusions. HG-ESS is more aggressive, characterized by high mitotic activity, necrosis, and genetic markers such as BCOR internal tandem duplication, often leading to advanced-stage diagnosis. Surgical resection is the cornerstone for managing early-stage ESS. A total hysterectomy with bilateral salpingo-oophorectomy (BSO) is recommended to prevent recurrence. Fertility-preserving approaches may be considered in LG-ESS but are associated with high recurrence rates. Lymphadenectomy is not routinely performed, given its limited prognostic value. HG-ESS, due to its aggressiveness, often requires additional treatment, including chemotherapy. Adjuvant therapy varies by subtype. LG-ESS responds well to hormonal treatments such as aromatase inhibitors and progestins, while tamoxifen is contraindicated. HG-ESS, lacking hormonal receptor expression, is managed with chemotherapy, often incorporating doxorubicin-based regimens. Radiotherapy may improve local control in select cases but shows limited impact on overall survival. Advanced-stage ESS treatment focuses on complete cytoreduction, supplemented by systemic therapies. Hormonal therapy remains the standard for advanced LG-ESS, whereas HG-ESS relies on chemotherapy. Prognosis depends on the subtype and stage. LG-ESS has favorable outcomes, with five-year survival exceeding 90% in early stages, but recurrent disease remains common. HG-ESS is associated with poorer survival due to its aggressive nature. Advances in molecular profiling offer promising avenues for personalized therapies, integrating genomic insights with targeted treatments to improve outcomes in this rare malignancy.

## Simple Summary

Endometrial stromal sarcoma is a rare malignant tumor arising from the endometrial stromal cells of the uterus. It is classified into low-grade and high-grade subtypes, which differ significantly in terms of histological features, clinical behavior, and treatment strategies. Due to its rarity and often non-specific clinical presentation, diagnosis may be delayed or incidental. This review provides a comprehensive overview of the clinical, pathological, and molecular characteristics of endometrial stromal sarcomas, highlighting current approaches to diagnosis, staging, and treatment. Surgical resection remains the cornerstone of management, while adjuvant therapies such as hormonal treatment or chemotherapy are considered based on tumor grade and receptor status. Recent advances in molecular profiling have contributed to a better understanding of the disease and may support the development of targeted therapies. This review aims to support the optimization of diagnostic and therapeutic pathways by synthesizing current evidence and clinical practice regarding this rare uterine neoplasm.

## 1. Introduction

Endometrial stromal sarcomas (ESS) are rare uterine malignancies that account for a small fraction of all uterine cancers but pose significant diagnostic and therapeutic challenges due to their heterogeneous biological behavior. Recent advances in histopathological classification and molecular profiling have improved our understanding of the distinct subtypes of ESS, particularly low-grade (LG-ESS) and high-grade (HG-ESS), which differ substantially in clinical course, prognosis, and treatment response. This narrative review aims to provide an updated overview of the clinical, pathological, and molecular landscape of ESS, with a focus on current diagnostic standards, therapeutic strategies, and emerging targeted approaches.

## 2. Methods

This review was conceived as a focused, expert-driven narrative synthesis rather than a systematic review. Therefore, it does not follow the PRISMA (Preferred Reporting Items for Systematic Reviews and Meta-Analyses) guidelines. The primary aim was to integrate and critically discuss the current knowledge on the clinical, pathological, and molecular characteristics of endometrial stromal sarcoma (ESS), including recent advances in classification and treatment.

To ensure methodological rigor, we conducted a structured literature search using the PubMed and Scopus databases. The search strategy included combinations of the following terms: “endometrial stromal sarcoma”, “low-grade ESS”, “high-grade ESS”, “BCOR”, “JAZF1”, “targeted therapy”, “molecular alterations”, and “immunotherapy”. We limited our search to peer-reviewed publications in English, published between January 2008 and December 2023. Studies eligible for inclusion comprised original research articles, narrative or systematic reviews, expert consensus guidelines, and clinically relevant retrospective series. Case reports, non-English articles, and preprints were generally excluded unless they contributed novel or clinically relevant insights.

## 3. Results

The initial search retrieved approximately 485 publications. After title and abstract screening, 178 articles were selected for full-text evaluation based on relevance to the scope of this review. Of these, 123 publications were ultimately included and cited in the manuscript.

Among the included sources:

- Sixty-two were original research articles (including retrospective and prospective cohort studies, molecular profiling studies, and clinical trials),
- Forty-one were review articles or meta-analyses,
- Twelve were expert consensus statements or clinical guidelines,
- Eight were selected case reports or case series offering unique diagnostic or therapeutic insights.

The included literature covers both low-grade and high-grade ESS, with particular attention to histopathological subtypes, immunohistochemical and genetic features, treatment strategies, and evolving therapeutic targets.

## 4. Discussion

### 4.1. Definition, Epidemiology, and Clinical Features

The most prevalent form of ESS is LG-ESS, which the 2020 World Health Organization (WHO) classification defines as “a malignant stromal tumor with cells that resemble proliferative-phase endometrial stroma, showing infiltrative growth with or without lymphovascular invasion”.

By contrast, HG-ESS is a far less common entity, described as having “uniform, high-grade, round, or spindle-shaped cells, sometimes featuring a low-grade component” [1].

While the majority of cases are diagnosed in women aged 40–55 (with a mean age of 50 years), the tumor can develop over a wide age range—from 16 to 83 years in LG-ESS and from 14 to 71 years in HG-ESS. More than half of the patients are premenopausal women, and those diagnosed with HG-ESS with a specific BCOR alteration (internal tandem duplication (ITD)) tend to be younger, with a mean age of 44 years. Risk factors for developing ESS include previous pelvic radiation, long-term estrogen or tamoxifen use, and, in some cases, a history of polycystic ovarian syndrome [2,3,4].

### 4.2. Clinical Presentation

Typical symptoms of ESS include abnormal uterine bleeding, pelvic discomfort, and painful menstruation (dysmenorrhea). In some cases, a uterine mass may be identified. Interestingly, about 25% of patients may remain asymptomatic. When symptoms do occur, they can result from the disease spreading beyond the uterus, a phenomenon seen in up to one-third of cases. This is particularly common in HG-ESS, where 70% of patients are diagnosed at an advanced stage. During a pelvic examination, a uterine or pelvic mass is often found, and in cases of LG-ESS, an endometrial polyp might be discovered during a standard gynecological check-up [5,6,7,8,9,10,11].

### 4.3. Diagnosis and Staging

ESS diagnosis remains challenging, especially in younger patients, due to the lack of a definitive imaging method. No single modality—ultrasound, MRI, CT, or PET-CT—has the specificity needed for an accurate diagnosis. Since ESS originates in the endometrium, histological examination remains the gold standard for diagnosis. Endometrial biopsy through hysteroscopy or uterine curettage is frequently employed. Final confirmation often occurs postoperatively after procedures like myomectomy or hysterectomy. When immediate surgery or biopsy is not possible, a core needle biopsy, guided by imaging, may be performed at specialized centers [12,13,14,15,16].

Typically, ultrasound identifies ESS as a hypoechoic, irregular mass originating in the endometrium. This finding can resemble other conditions, such as adenomyosis or fibroids undergoing degeneration. In some instances, the endometrium may also appear heterogeneous or exhibit cystic degeneration [17,18,19].

**Figure 1:**
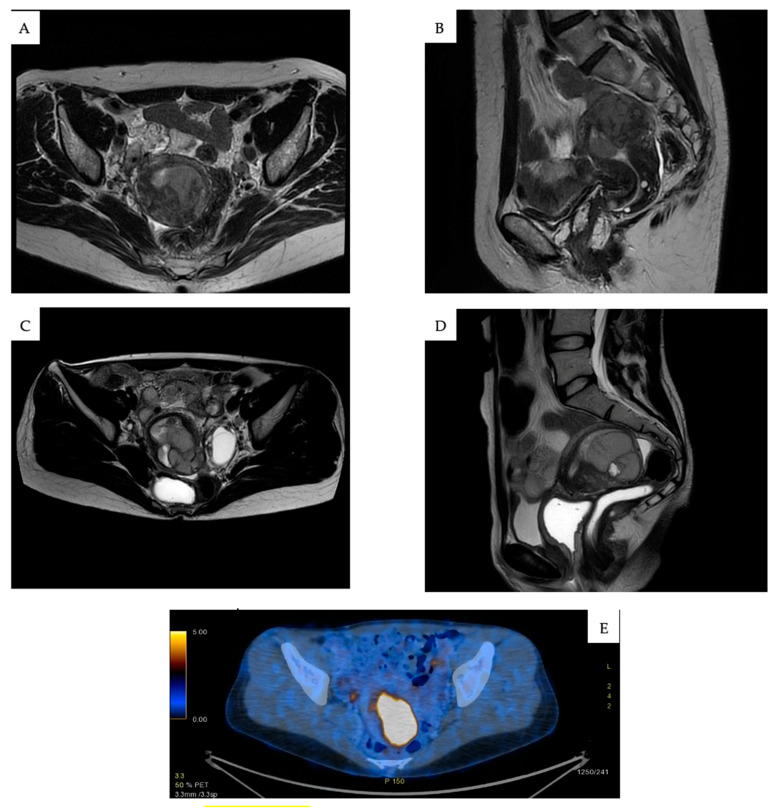
(**A**) Axial pelvic MRI image showing a hypointense endometrial mass with infiltrative margins in a patient with low-grade ESS (LG-ESS). (**B**) Sagittal pelvic MRI image highlighting endometrial thickening and myometrial infiltration in LG-ESS. (**C**) Axial MRI scan demonstrating extensive myometrial invasion and central necrosis in a case of high-grade ESS (HG-ESS). (**D**) Coronal MRI image depicting heterogeneous signal intensity with hemorrhagic and necrotic areas in HG-ESS. (**E**) PET-CT scan showing elevated FDG uptake in a metabolically active HG-ESS lesion. All figures are original and created by the authors.

MRI is frequently employed to detect ESS, which often presents as an endometrial mass, with either smooth or irregular contours (Figure 1A,B). Myometrial infiltration is a common feature, particularly in HG-ESS, which is more prone to displaying hemorrhage and extensive necrotic regions (Figure 1C,D) [20,21,22]. Unlike endometrial cancer, ESS often manifests as multiple nodules infiltrating the myometrium [21]. PET-CT may aid in assessing metabolic activity, although it lacks specificity for definitive diagnosis (Figure 1E). LG-ESS, due to its slow growth, generally demonstrates lower FDG uptake compared to HG-ESS or leiomyosarcomas [23]. There are no serum tumor markers specific to ESS.

A full diagnostic approach involves imaging beyond the pelvis. A CT scan is typically performed to evaluate the potential spread to distant sites, while a pelvic MRI is essential for assessing the extent of local invasion or residual disease; depending on the clinical scenario, PET-CT may also be employed.

The FIGO 2009 staging (Table 1) system is currently used to stage ESS and remains a critical factor in determining prognosis [24].

### 4.4. Histopathological and Molecular Characteristics

#### 4.4.1. A. Low-Grade ESS (LG-ESS)

**Figure 2:**
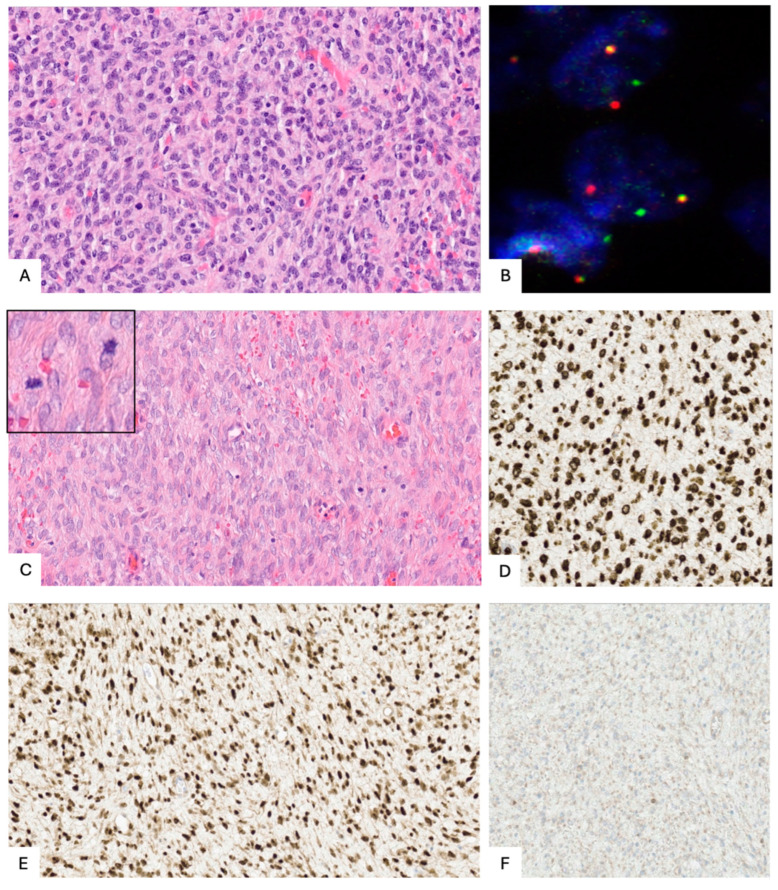
(**A**) Low-grade ESS (LG-ESS) showing proliferation of uniform oval to spindle-shaped cells with minimal cytologic atypia and low mitotic activity (H&E, ×20). (**B**) Fluorescence in situ hybridization (FISH) for JAZF1 gene rearrangement in LG-ESS: split signals of 3′ (red) and 5′ (green) probes indicate gene rearrangement; yellow dot represents normal spot without gene rearrangement. (**C**) High-grade ESS (HG-ESS) with BCOR internal tandem duplication: proliferation of oval to spindle-shaped cells with high mitotic index (H&E, ×20). (**D**) Diffuse nuclear BCOR immunoreactivity in HG-ESS. (**E**) Strong and diffuse nuclear staining for cyclin D1 in HG-ESS. (**F**) CD10 expression is absent or markedly reduced in HG ESS with BCOR alterations. All figures are original and created by the authors.

LG-ESS is composed of densely packed islands of endometrial stromal-like cells that infiltrate the myometrium in a tongue-like pattern. This pattern often extends more than three finger-like projections measuring more than 3 mm from the tumor’s edge and is associated with lymphovascular space invasion (LVSI) [15]. These cells typically exhibit minimal atypia, with necrosis rarely present. Mitotic activity is low, typically fewer than 10 mitoses per 10 high-power fields (Figure 2A). The cells express CD10, estrogen receptors (ER), and progesterone receptors (PR) diffusely, while cyclin D1 positivity is focal. Smooth muscle markers are usually negative, although in cases of smooth muscle metaplasia, desmin and H-caldesmon may be positive [1].

Genetic alterations such as JAZF1, PHF1, or EPC1 gene fusions are often identified, with JAZF1-SUZ12 being the most common (Figure 2B). Nonetheless, the absence of such rearrangements does not exclude the diagnosis of LG-ESS. Histone deacetylase has been investigated as a potential prognostic marker and therapeutic target, highlighting its potential, which may lead to new opportunities for targeted therapies in the treatment of the disease [25].

It is noteworthy that, on rare occasions, low-grade ESS with JAZF1 and PHF1 rearrangements can transform into high-grade ESS either at the time of diagnosis or during recurrence. This transformation may be signaled by increased mitotic activity, enlarged nuclei, or prominent nucleoli, indicating more aggressive behavior [26].

#### 4.4.2. B. High-Grade ESS (HG-ESS)

HG-ESS is distinguished by high-grade cells that are either round or spindle-shaped, sometimes featuring a low-grade component. These tumors are known for their expansive, infiltrative, or permeative growth patterns. Necrosis is commonly observed, along with a high mitotic rate (greater than 10 mitoses per 10 HPF) and frequent lymphovascular invasion (LVSI) [1]. HG-ESS can be divided into several categories based on morphology, immunohistochemical markers, FISH, or targeted RNA sequencing:

#### 4.4.3. HG-ESS with YWHAE-NUTM2A/B Fusion

This subtype typically features round cells with eosinophilic cytoplasm and high-grade nuclei with irregular edges. In some cases, a fibromyxoid or low-grade endometrial stromal component may also be seen. CD10 and hormone receptors are usually negative but may show positivity in low-grade regions. Cyclin D1 is often strongly positive, expressed in over 70% of cells, while C-Kit may be positive, though DOG1 remains negative. BCOR staining is frequently positive, even in the absence of BCOR gene alterations [27].

#### 4.4.4. HG-ESS with BCOR Alterations (Including BCOR or BCORL1 Fusions or BCOR Internal Tandem Duplication (ITD))

Tumors in this group generally have spindle or oval-shaped cells within a myxoid background, which can mimic myxoid leiomyosarcoma. In BCOR or BCORL1 fusion cases, cyclin D1 positivity is common, although BCOR expression is observed in only about 50% of cases. CD10 is generally positive, with varying levels of ER and PR expression. In BCOR ITD tumors, cyclin D1 and BCOR are diffusely positive, but CD10 expression is reduced, and ER and PR are typically negative [28] (Figure 2C–F).

#### 4.4.5. ESS with KAT6B/A::KANSL1 Fusion

This newly identified subtype resembles low-grade ESS in its morphology, though it may follow a more aggressive clinical course, even without extensive invasive growth. More case studies are needed to fully understand its behavior and clinical significance [29].

#### 4.4.6. HG-ESS Not Otherwise Specified (NOS)

This category refers to high-grade sarcomas that contain a low-grade component, which may or may not be associated with JAZF1 fusions, and often show signs of dedifferentiation [30].

**Figure 3:**
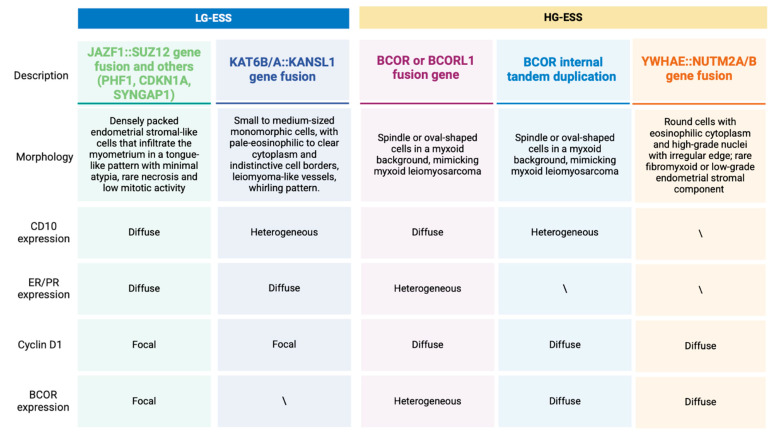
Summary of the morphological and immunohistochemical characteristics of endometrial stromal sarcoma subtypes. All figures are original and created by the authors.

Diagnostic tools such as immunohistochemistry for CD10, hormone receptors, cyclin D1, and BCOR, along with testing for gene fusions or BCOR ITD, can assist in confirming the diagnosis (Figure 3). It remains unclear whether these various HG-ESS subtypes differ in prognosis or treatment outcomes [1,31].

## 5. Surgical Management of Early-Stage ESS

Patients with ESS, which is part of the broader category of soft tissue sarcomas, should be treated at specialized centers to ensure the best possible prognosis [32]. For early-stage ESS, the preferred treatment involves complete surgical removal of the tumor with clear margins. A total hysterectomy with bilateral salpingo-oophorectomy (BSO) is recommended, ensuring the uterus is removed intact, without morcellation, to mitigate the risk of tumor cell dissemination. While laparotomy is the most common approach, minimally invasive techniques may be used if the uterus can be removed intact [12,13,14,15,16,31].

Lymphadenectomy is not generally recommended, as lymph node involvement does not seem to affect prognosis significantly. Instead, it should be limited to patients with visible nodal disease on preoperative imaging or intraoperative findings [12,13,14,15,16,33,34,35,36,37,38].

When there is no visible adnexal tumor or gross pelvic extra-uterine disease, the likelihood of ovarian metastasis is low [15]. A meta-analysis of 17 studies showed that patients with LG-ESS who preserved their ovaries had a higher risk of recurrence compared to those who underwent bilateral salpingo-oophorectomy (BSO) (46.8% versus 24.2%, OR = 2.70, 95% CI = 1.39–5.28). However, there was no significant difference in mortality rates between the two groups (5.9% versus 7%, OR = 0.80, 95% CI = 0.18–3.47) [33,39]. These findings were supported by a study conducted by Nasioudis et al., which analyzed 743 premenopausal women with stage I LG-ESS from the National Cancer Database. The five-year overall survival (OS) rates for those who had BSO and those who preserved their ovaries were 96.2% and 97.1%, respectively, with no significant difference in OS even after adjusting for confounding factors (HR = 1.28, 95% CI = 0.51–3.19) [40]. Given the frequent absence of hormone receptor expression in HG-ESS, ovarian preservation may be considered for premenopausal women with early-stage HG-ESS [12,15]. In a retrospective study of six patients with HG-ESS who opted for ovarian preservation (four of whom were stage I), no recurrences were observed during follow-up (60–71 months) [41].

In cases where ESS is discovered following a hysterectomy or after morcellation, additional imaging is required to evaluate for any remaining disease. If residual tumor is detected or prior treatment was incomplete, a second surgery may be necessary [13,15].

### Fertility-Sparing Treatment

In young women with LG-ESS who wish to preserve their ability to have children, fertility-sparing approaches have been investigated in case of LG-ESS in women of childbearing age. These methods typically rely on hormone-based treatments, including GnRH analogs, progestins, or the use of a levonorgestrel-releasing intrauterine device (IUD). While successful pregnancies have been reported, the recurrence risk remains substantial, with some studies indicating relapse rates exceeding 50% [42].

A series of 153 cases of LG-ESS, including 19 patients treated with fertility-sparing treatment (FST) through myomectomy, showed a recurrence rate of 78.9%, compared to 25.4% in those who underwent hysterectomy (*p* = 0.0075), with an average recurrence interval of 20.5 months. One case of death due to disease was reported following FST [43]. Another report involving 17 cases of LG-ESS treated with FST found a recurrence rate of 58.8%, with no deaths recorded [44]. This underscores the need for careful follow-up and eventual radical surgery once childbearing is complete.

In cases of HG-ESS, fertility-sparing treatments are not recommended due to the aggressive nature of the disease [12,15,44,45,46,47,48].

## 6. Adjuvant Treatment

### 6.1. Low-Grade Endometrial Stromal Sarcoma (LG-ESS)

The role of adjuvant therapy in LG-ESS remains a subject of debate, largely due to the absence of robust prospective studies directly addressing its efficacy [49]. Nevertheless, hormonal treatments are widely utilized, given the strong expression of estrogen (ER) and progesterone receptors (PR) in these tumors. Common options include aromatase inhibitors (AIs) and progestins like megestrol acetate or medroxyprogesterone acetate, which are often employed in adjuvant, recurrent, or metastatic disease settings [50]. In contrast, tamoxifen is contraindicated because of its estrogen-like effects on the endometrium, which may promote tumor progression [51]. The paucity of definitive data highlights the need for further studies to optimize treatment strategies in these cases [15].

AIs are often favored due to their relatively better side effect profile and long-term tolerability. They have also been found effective after progestin therapy failure [50]. Although no optimal duration for adjuvant therapy has been established, recommendations suggest treatment lasting at least two years, extending up to five years based on data from breast cancer management [52].

In a cohort of 43 patients with ESS (no data on grade), Chu et al. found that continuous progestin therapy led to lower recurrence rates compared to observational management in stage I disease (14.3% vs. 38.5%, *p* = 0.26) and across all stages (33% vs. 50%, *p* = 0.38) [53]. Amant et al. reported a significant improvement in disease-free survival (DFS) with hormonal adjuvant treatment in 31 patients with stage III–IV LG-ESS. Similarly, Chu et al. found a reduced risk of relapse in 22 LG-ESS patients receiving progestins (31% vs. 67%) [54]. Comert et al. described a series of 37 LG-ESS patients who received various adjuvant therapies (radiotherapy, chemotherapy, or progestins) and found that only hormone therapy was linked to a reduced recurrence rate (0% vs. 38.5% in the surgery-only group), even for stage I patients [55]. However, Leath et al. reported no statistically significant difference in overall survival (OS) between 30 patients treated with adjuvant progestins and those under observation (94 months vs. 72 months, *p* = 0.07) [56]. A meta-analysis of 10 studies showed that hormonal treatment improved DFS for stage I–II LG-ESS (*p* = 0.02) and OS for stages III–IV. Currently, chemotherapy is not indicated for LG-ESS, as no evidence supports its use [57].

The use of adjuvant radiotherapy in ESS has been investigated in several retrospective studies, showing a positive effect on local control, though no improvement in overall survival (OS) was observed [58,59]. However, in many cases, data on tumor grade were missing. Wang et al. reported a lower rate of local recurrence following radiotherapy (RT) in a series of 152 women with stage I–II LG-ESS [60]. Contrarily, a SEER analysis involving 1010 women with ESS found no OS benefit associated with adjuvant RT [61,62]. The only randomized trial on pelvic radiation for uterine sarcomas, conducted by the European Organization for Research and Treatment of Cancer (EORTC), did not show any advantage in progression-free survival (PFS) or OS for patients with stage I–II high-grade uterine sarcoma, though 30 ESS patients were included without specific subgroup analysis [63].

### 6.2. High-Grade Endometrial Stromal Sarcoma (HG-ESS)

The role of adjuvant chemotherapy in HG-ESS remains uncertain, as there are no randomized controlled trials (RCTs) to establish its efficacy. In a retrospective analysis of 1383 women with high-grade ESS (HG-ESS) from the National Cancer Database (NCDB), adjuvant chemotherapy was linked to improved overall survival (OS) (*p* < 0.001), with even a modest benefit observed in early-stage cases [64]. A French retrospective study of 9 stage I–II and 21 stage III–IV patients showed no statistically significant survival benefit from adjuvant chemotherapy with Adriamycin and ifosfamide [65]. However, a multivariate analysis of 39 patients with localized HG-ESS and undifferentiated uterine sarcoma (UUS) in the French Sarcoma Group database revealed a significant survival advantage for those who received adjuvant chemotherapy (41 months vs. 10.3 months without it) [66]. For patients with stage I HG-ESS who have undergone complete surgical resection, observation may be sufficient, but adjuvant chemotherapy is considered in cases with high-risk features, such as large tumor size, high mitotic index, or surgical morcellation. In more advanced stages, a combination of chemotherapy (doxorubicin or epidoxorubicin with or without Ifosfamide) and external beam radiotherapy (EBRT) may be considered to improve outcomes [6,12,13,14,15,16].

There is limited evidence regarding the use of radiotherapy in HG-ESS. Retrospective studies suggest that while radiotherapy may improve local control, it has not been shown to significantly impact overall survival. Therefore, its use remains reserved for select cases with specific risk factors, such as tumor size, positive margins and/or number of positive lymph nodes removed, and local involvement (cervical, parametrial, vaginal, serosal). Most of the available data come from retrospective studies with varied patient groups. The only randomized phase III trial (EORTC 55874) evaluated the impact of adjuvant pelvic radiotherapy compared to observation on pelvic relapse in 224 patients with surgically treated stage I/II uterine sarcoma. While pelvic radiotherapy improved local control in the broader cohort, no benefit in local relapse or overall survival (OS) was found in the subgroup of leiomyosarcoma patients (n = 99) [63]. No specific analysis was conducted for HG-ESS. Retrospective studies generally show improved local pelvic control with radiotherapy but no clear OS benefit. Two studies from the French Sarcoma Group reported better disease-free survival (DFS) and OS in HG-US patients who underwent adjuvant radiotherapy after complete resection [65,66]. Similar findings were noted in a retrospective analysis involving HG-ESS and undifferentiated uterine sarcoma (USS) patients from the NCDB and the Rare Cancer Network [67]. However, other retrospective studies have failed to demonstrate a survival advantage with postoperative radiotherapy [68,69,70]. A structured overview of adjuvant therapeutic strategies for both LG-ESS and HG-ESS is provided in the Supplementary Materials (Figures S1 and S2).

## 7. Treatment of Advanced and Recurrent ESS

In advanced stages of ESS, the primary objective is complete surgical cytoreduction, aiming to remove all visible disease. Achieving complete resection has been shown to significantly improve survival outcomes, and leaving residual disease after surgery is associated with a poor prognosis [56,65]. For patients in whom complete surgery is not possible, systemic therapy (hormonal or chemotherapy) is typically administered, potentially followed by secondary surgery if the tumor responds well. External beam radiotherapy (EBRT) and/or focused percutaneous radiotherapy may be options for treating extra-abdominal disease or in palliative care situations [59,71]. The potential of immunotherapy is currently being studied [72].

### 7.1. LG-ESS

In cases of advanced or recurrent LG-ESS, hormonal therapy is widely regarded as the cornerstone of treatment. Commonly employed options include aromatase inhibitors (AIs), progestins (such as megestrol acetate or medroxyprogesterone acetate), GnRH analogs, and Fulvestrant. These therapies leverage the hormone-sensitive nature of LG-ESS, which often exhibits strong estrogen and progesterone receptor expression [12,73,74,75,76,77].

In some scenarios, particularly when the disease appears potentially resectable, clinicians may choose to begin with hormonal therapy to evaluate the tumor’s responsiveness. This approach can help determine whether surgical intervention is feasible or beneficial, reducing the extent of surgery required in certain cases [12].

Hormonal therapy remains the cornerstone of management for advanced or recurrent LG-ESS, with evidence of durable responses even in second-line settings. This supports its versatility and the need to tailor treatment to individual response [73,74,75,76,77]. Despite their benefits, however, the choice of hormonal agent and sequencing of treatments remain areas where further research is needed to optimize outcomes [12].

In various retrospective studies, aromatase inhibitors (AIs) and progestins have demonstrated a high response rate and effective long-term disease control in advanced or recurrent cases [52,76]. Even when progression occurs after initial hormonal therapy, second-line hormonal treatments have been shown to provide sustained disease stability [52]. Other hormonal options include fulvestrant and GnRH analogues, with fulvestrant currently being tested in the FUCHSia Trial [78]. In some cases, de novo ESR1 hotspot mutations, often seen with histologic high-grade transformation or altered estrogen receptor expression, have been linked to resistance to aromatase inhibitors [79]. Rather than switching to chemotherapy, the ESGO-EURACAN-GCIG guidelines recommend treatment with estrogen receptor degraders, such as fulvestrant or newer-generation estrogen receptor degraders, as an alternative. Further research is needed to fully understand these mutations and optimize treatment [15].

### 7.2. HG-ESS

HG-ESS, by contrast, typically requires chemotherapy in cases of advanced or recurrent disease, as hormonal treatments are ineffective. The goal remains complete cytoreduction when possible. When complete cytoreduction is not feasible, primary chemotherapy should be administered, with debulking surgery considered if a favorable clinical and radiological response is achieved [80]. Some experts suggest administering chemotherapy prior to surgery, even in cases where the disease is initially resectable, as a way to assess the tumor’s chemosensitivity [12]. Multi-agent chemotherapy regimens, often involving anthracyclines like doxorubicin, ifosfamide, or other soft tissue sarcoma agents (e.g., gemcitabine or trabectedin), are frequently employed. Hyperthermic intraperitoneal chemotherapy (HIPEC) may be considered in clinical trial settings, as it is not yet established as standard care [68,81,82].

Chemotherapy trials for advanced and metastatic uterine sarcomas seldom include patients with HG-ESS. However, the chemotherapy regimens used for HG-ESS are typically the same as those employed for undifferentiated uterine sarcomas (UUS) and other soft tissue sarcomas. The EORTC phase III randomized trial compared doxorubicin alone to doxorubicin combined with ifosfamide and pegfilgrastim in patients with locally advanced, unresectable, or metastatic soft tissue sarcomas (STSs). The combination therapy showed a higher response rate (26% vs. 14%, *p* < 0.0006) and improved progression-free survival (PFS) (7.4 vs. 4.6 months), but it also led to a higher incidence of grade 3–4 hematological toxicities and did not improve overall survival (OS) [83]. In a phase 2 trial conducted by Pautier et al., trabectedin combined with doxorubicin was investigated as a first-line treatment for advanced uterine or soft-tissue leiomyosarcoma (LMS). The study found that this combination therapy yielded promising results, with an overall response rate of 37% and a median progression-free survival (PFS) of 12.1 months. Also in this case, the treatment was associated with significant toxicities, including neutropenia and elevated liver enzymes, highlighting the need to balance efficacy with safety in this patient population [84]. The same authors studied, in a phase 3 trial, the combination of doxorubicin and trabectedin, followed by trabectedin alone, which was compared to doxorubicin monotherapy in patients with metastatic or unresectable leiomyosarcoma. The study found no significant improvement in overall survival (OS) with the combination therapy, though progression-free survival (PFS) was slightly longer in the combination group [85].

Hormonal therapy is not effective in the treatment of HG-ESS. However, in HG-ESS cases with BCOR alterations that express estrogen receptors (ER), especially in metastatic or recurrent disease, a combination of an aromatase inhibitor (AI) with a CDK 4/6 inhibitor may be a treatment option [86].

## 8. Prognosis of Endometrial Stromal Sarcoma

### 8.1. Low-Grade ESS (LG-ESS)

**Figure 4:**
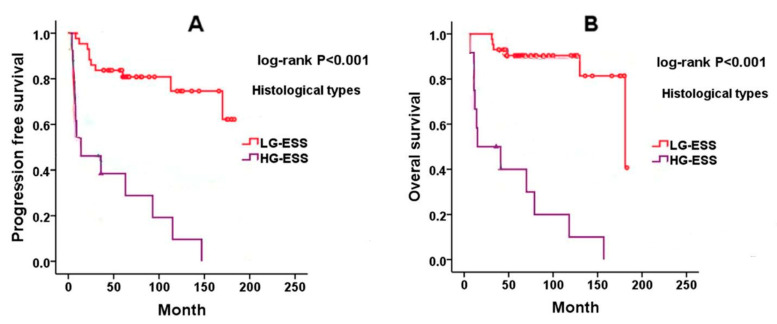
Kaplan–Meier survival curves comparing progression-free survival (**A**) and overall survival (**B**) between low-grade endometrial stromal sarcoma (LG-ESS) and high-grade endometrial stromal sarcoma (HG-ESS), adapted from Wang et al., BMC Cancer, 2022 [87]. LG-ESS shows significantly improved prognosis over HG-ESS (log-rank *p* < 0.001 for both outcomes).

LG-ESS typically follows an indolent course, with relatively favorable long-term outcomes even in the presence of recurrent disease. The most important prognostic factor is the stage at diagnosis [7]. Five-year survival rates exceed 90% for patients with stage I–II disease, though they drop to around 50% for those with stage III–IV [19]. While about 60% of patients present with stage I disease, only 20% are diagnosed at stage IV. Late recurrences are frequently observed, even in early-stage disease, highlighting the need for prolonged surveillance. While most relapses occur within the pelvis and abdomen, distant sites such as the lungs and vagina may also be involved. Positive hormone receptor status (ER and PR) is associated with better overall survival [12,14,15]. A direct comparison between LG-ESS and HG-ESS in terms of both progression-free survival (PFS) and overall survival (OS) is illustrated in Figure 4, derived from previously published literature [87]. However, the prognostic significance of mitotic activity, tumor size, and cytologic atypia in early-stage LG-ESS remains controversial.

On rare occasions, LG-ESS may transform into a high-grade tumor, which is associated with markedly poorer prognosis [26].

### 8.2. High-Grade ESS (HG-ESS)

HG-ESS is a far more aggressive tumor, with a prognosis that resembles undifferentiated uterine sarcomas (UUS). Recurrences tend to occur early—often within a year of diagnosis—and patients are at higher risk of death from the disease. The prognosis is particularly poor in patients diagnosed at advanced stages [65,66].

Tumor size and stage are critical prognostic factors, with larger tumors and higher stages associated with a significantly increased risk of death. Studies have shown that tumors with high mitotic activity and extensive necrosis are also associated with worse outcomes [88,89].

Certain genetic alterations, such as ZC3H7B-BCOR, EPC1-SUZ12, and EPC1-BCOR fusions, are linked to more aggressive behavior and poorer survival rates in HG-ESS patients [90].

## 9. Conclusions and Future Perspectives

Recent advances in molecular diagnostics have highlighted the crucial role of next-generation sequencing (NGS) in refining the classification and clinical management of endometrial stromal sarcomas (ESS) [91]. Integrating morphological, immunohistochemical, and molecular profiling—particularly through the identification of recurrent gene fusions such as JAZF1-SUZ12, YWHAE-NUTM2, and ZC3H7B-BCOR—offers a more precise diagnostic framework and enables stratification of patients for tailored therapeutic approaches.

In high-grade ESS (HG-ESS), comprehensive genomic and transcriptomic analyses have revealed actionable alterations in pathways such as homologous recombination repair, PI3K/AKT/mTOR signaling, and cell cycle regulation. Additionally, subsets of HG-ESS display increased immune infiltration and overexpression of immune-related genes, suggesting a potential sensitivity to immunotherapeutic strategies. These molecular features, including PD-L1 expression and high tumor mutation burden in selected cases, support the rationale for incorporating immune checkpoint inhibitors into future clinical trials [92]. Recent data also suggest that specific gene alterations, such as MEGF8 and SETD1B mutations, may be associated with increased immunotherapy responsiveness and could represent novel predictive biomarkers or therapeutic targets in gynecologic sarcomas, including subsets of ESS [72].

As such, molecular characterization is anticipated to play an increasingly central role in guiding precision oncology approaches for ESS. Future research should focus on validating predictive biomarkers, elucidating mechanisms of therapeutic resistance, and exploring targeted agents and immunotherapies in well-defined molecular subgroups of ESS.

## Figures and Tables

**Table 1:** Table showing the FIGO 2009 staging classification for endometrial stromal sarcoma based on tumor extent and spread.

Stage	Description
I stage	Tumor confined to the uterus
IA	Tumor limited to endometrium/endocervix stroma with no myometrial invasion
IB	Less than or equal to half myometrial invasion
IC	More than half myometrial invasion
II stage	Tumor extends to the pelvis
IIA	Involvement of adnexa (ovaries or fallopian tubes)
IIB	Tumor extends to extrauterine pelvic tissue
III stage	Tumor infiltrates abdominal tissues (not just protruding into the abdomen)
IIIA	One site
IIIB	More than one site
IIIC	Metastasis to pelvic and/or para-aortic lymph nodes
IV stage	Tumor invades bladder/rectum or shows distant metastasis
IVA	Direct invasion of bladder and/or rectum
IVB	Distant metastases (e.g., lungs, liver, bones)

## Data Availability

Data sharing not applicable to this article as no datasets were generated or analyzed.

## Supplementary Materials

The following supporting information can be downloaded at: https://www.mdpi.com/article/10.3390/cancers17111893/s1, Figure S1. Treatment algorithm for LG-ESS; Figure S2. Treatment algorithm for HG-ESS.
