# Supplementary material for: Endometrial Stromal Sarcoma: An Update
Source: Cancers (Basel). 2025 Jun 5;17(11):1893. doi: 10.3390/cancers17111893 (PMC12153635; doi:10.3390/cancers17111893)
Supplement: Supplementary file 1 [file cancers-17-01893-s001.zip › cancers-3633273-supplementary.pdf]

Flowchart ESS

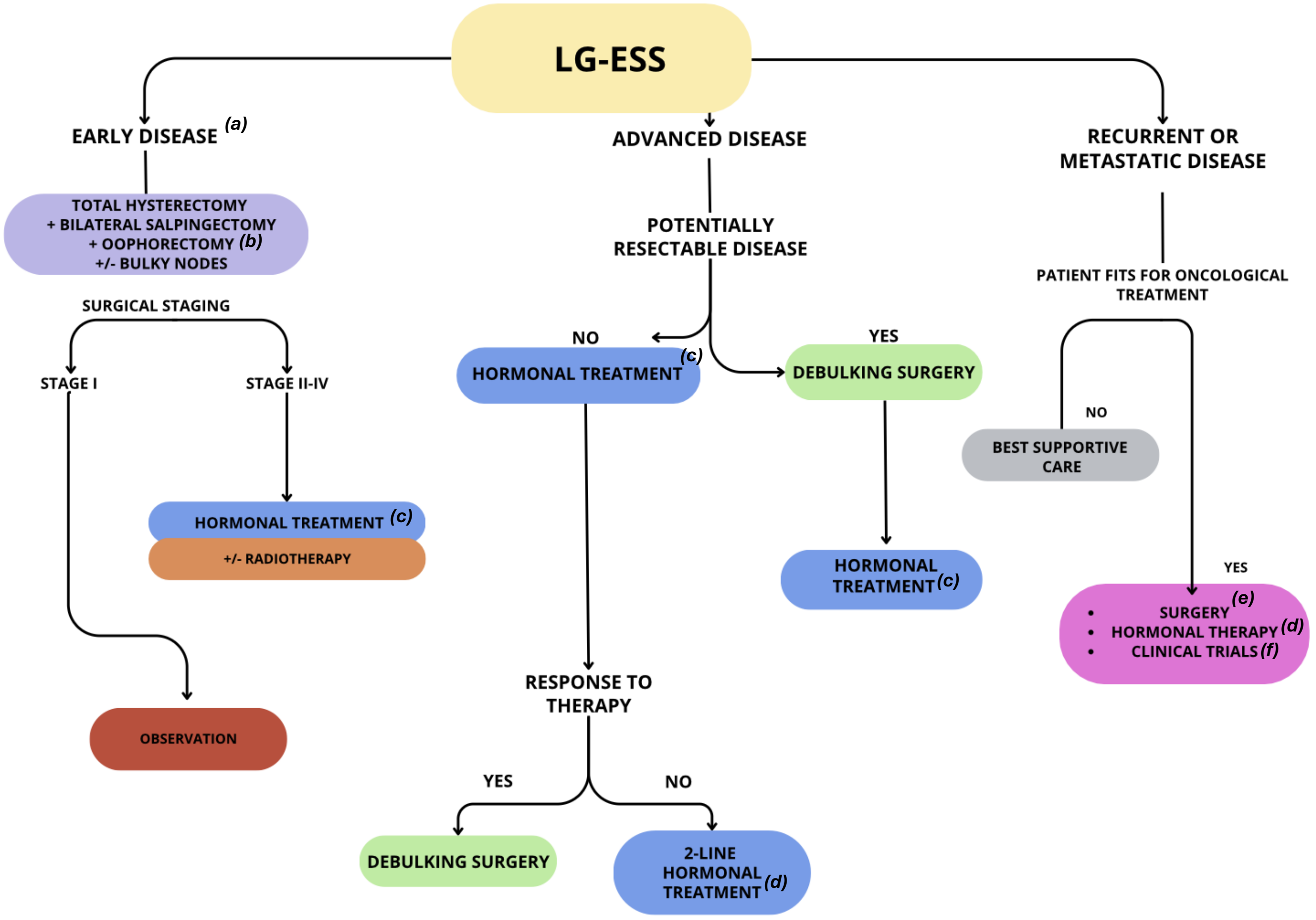

Figure S1. Treatment algorithm for LG-ESS

a) Fertility sparing treatment (FST) (myomectomy/polypectomy) cannot be considered as standard treatment and should be considered as experimental, in highly motivated young women and after appropriate counselling.
b) It may be omitted in young premenopausal women whose ovaries appear unaffected macroscopically, after careful discussion.
c) Aromatase inhibitors (Letrozole), progestins (Megestrol acetate or Medroxyprogesterone acetate).
d) Aromatase inhibitors (Letrozole) or progestins (Megestrol acetate or Medroxyprogesterone acetate), Gonadotropin-Releasing Hormone analogue (Leuprolide).
e) In oligometastatic disease; it should be considered if complete resection is possible with acceptable morbidity.
f) Selective estrogen receptor degrader (Fulvestrant) (NCT03926936)

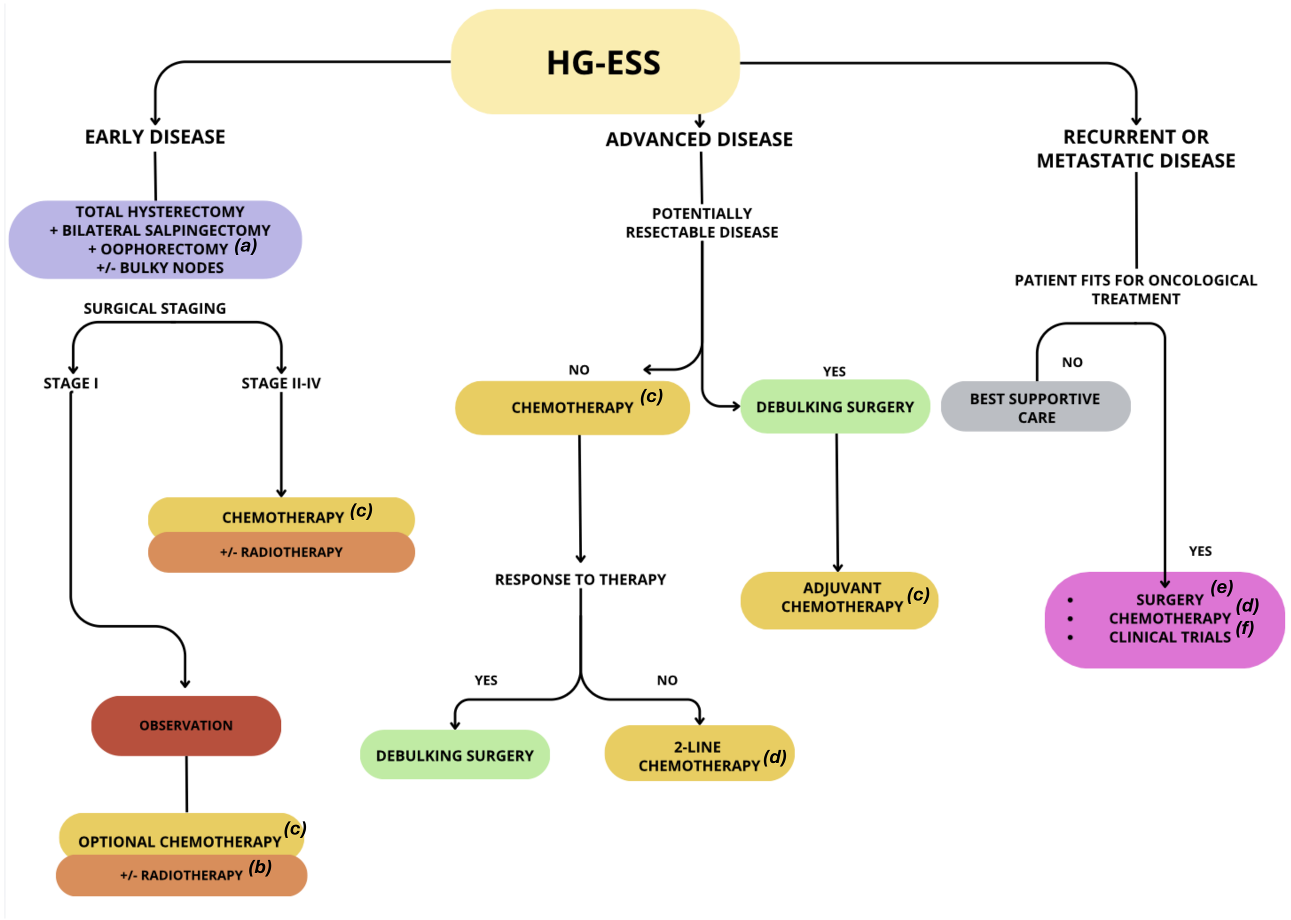

Figure S2. Treatment algorithm for HG-ESS

a) It may be omitted in young premenopausal women whose ovaries appear unaffected macroscopically.
b) In selected cases.
c) Doxorubicin or Epidoxorubicin with or without Ifosfamide.
d) Gemcitabine or Trabectedin.
e) In oligometastatic disease; it should be considered if complete resection is possible with acceptable morbidity.
f) Anti-PD-1 (programmed death-1) monoclonal antibody Nivolumab (NCT03241745).
